# Gut–Vaginal Microbiome Crosstalk in Ovarian Cancer: Implications for Early Diagnosis

**DOI:** 10.3390/pathogens14070635

**Published:** 2025-06-25

**Authors:** Hao Lin, Zhen Zeng, Hong Zhang, Yongbin Jia, Jiangmei Pang, Jingjing Chen, Hu Zhang

**Affiliations:** 1 Department of Gastroenterology, West China Hospital, Sichuan University, 37 Guoxue Lane, Chengdu 610041, China; 2019181620268@stu.scu.edu.cn (H.L.); 2020324025194@stu.scu.edu.cn (Z.Z.); 2022324025194@stu.scu.edu.cn (H.Z.); 18683576069@163.com (J.P.); jingjingchen0919@163.com (J.C.); 2 Centre for Inflammatory Bowel Disease, West China Hospital, Sichuan University, Chengdu 610041, China; 3 Lab of Inflammatory Bowel Disease, Frontiers Science Center for Disease-Related Molecular Network, West China Hospital, Sichuan University, Chengdu 610041, China; jiayongbin@wchscu.edu.cn

**Keywords:** ovarian cancer, gut, vagina, microbiomes, biomarkers

## Abstract

Ovarian cancer remains a formidable global health burden, characterized by frequent late-stage diagnosis and elevated mortality rates attributable to its elusive pathogenesis and the critical lack of reliable early-detection biomarkers. Emerging investigations into the gut–vaginal microbiome axis have unveiled novel pathogenic mechanisms and potential diagnostic targets in ovarian carcinogenesis. This comprehensive review systematically examines the compositional alterations in and functional interplay between vaginal and intestinal microbial communities in ovarian cancer patients. We elucidate three principal mechanistic pathways through which microbial dysbiosis may drive oncogenesis: (1) estrogen-mediated metabolic reprogramming via β-glucuronidase activity; (2) chronic activation of pro-inflammatory cascades (particularly NF-κB and STAT3 signaling); (3) epigenetic silencing of tumor suppressor genes through DNA methyltransferase modulation. We propose an integrative diagnostic framework synthesizing multi-omics data—incorporating microbial profiles, metabolic signatures, pathway-specific molecular alterations, established clinical biomarkers, and imaging findings—within a multifactorial etiological paradigm. This innovative approach aims to enhance early-detection accuracy through machine learning-enabled multidimensional pattern recognition. By bridging microbial ecology with tumor biology, this review provides novel perspectives for understanding ovarian cancer etiology and advancing precision oncology strategies through microbiome-targeted diagnostic innovations.

## 1. Introduction

Ovarian cancer persists as the most lethal gynecologic malignancy globally, with 324,398 new cases and 206,839 deaths reported in 2022 [1]. This dismal prognosis stems primarily from delayed detection, as over 60% of the patients present with advanced-stage disease (III/IV) due to nonspecific early symptoms, resulting in a 5-year survival rate of merely 30–50% [2,3]. Despite emerging immunotherapies such as PARP inhibitors (e.g., olaparib, rucaparib) and anti-angiogenic agents (e.g., bevacizumab), tumor cytoreduction combined with platinum–paclitaxel chemotherapy remains the frontline therapeutic strategy [4,5,6]. The stagnant therapeutic advancements over three decades underscore the urgent need to elucidate ovarian carcinogenesis mechanisms and identify novel diagnostic biomarkers.

**Figure 1:**
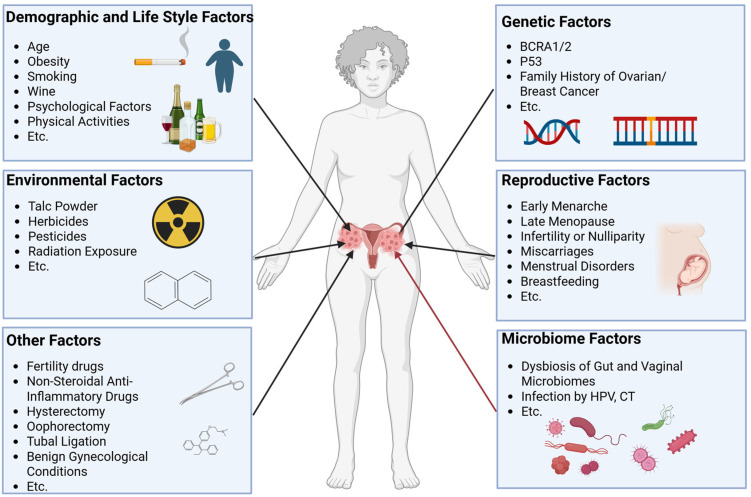
Associated factors of ovarian cancer. Associated factors of ovarian cancer have been reported and mainly include environmental factors, genetic factors, demographic and lifestyle factors, reproductive factors, microbiome factors, and other factors. These factors together change the risk of developing ovarian cancer.

The etiology of ovarian cancer involves multifactorial interactions, with genetic predisposition [7,8], environmental exposures [9], lifestyle factors [10], reproductive history [11], and emerging microbiome influences collectively contributing to it (Figure 1). Recent attention has focused on the microbiome–ovarian cancer axis, particularly its potential roles in disease initiation and progression.

Emerging evidence highlights the gut–vaginal microbiota axis as a critical interface in human health, characterized by symbiotic relationships and bidirectional communication [12,13,14,15,16,17,18,19]. Distinct microbial signatures in both gut and vaginal ecosystems have been identified in ovarian cancer patients [20], with mechanistic links proposed through estrogen metabolism [21], chronic inflammation [22], and epigenetic regulation [23]. These findings position microbial dysbiosis as a promising biomarker for early detection. In this review, we briefly summarize the characteristics of the gut and vaginal microbiomes and discuss the correlation between intestinal and vaginal microbiomes and ovarian cancer. We hope to provide a possible solution to prevention and early diagnosis for patients with ovarian cancer from the microbiome point of view.

This review systematically synthesizes the current evidence on intestinal and vaginal microbiome alterations in ovarian cancer, analyzes their pathophysiological interactions, and evaluates proposed mechanisms linking microbial dynamics to carcinogenesis. By delineating these relationships, we aim to advance the understanding of ovarian cancer etiology and inform innovative diagnostic strategies.

## 2. Characteristics of the Gut and Vaginal Microbiota in Ovarian Cancer

### 2.1. Characteristics of the Gut and Vaginal Microbiota in Healthy Women

**Gut microbiota:** The gut microbiota comprises several phyla, including *Bacteroidetes*, *Firmicutes*, *Actinobacteria*, *Proteobacteria*, and *Verrucomicrobia*. Among these, *Bacteroidetes* and *Firmicutes* are the two dominant phyla, typically constituting over 90% of the total microbiota, followed by *Actinobacteria*, which usually accounts for less than 5%. *Proteobacteria* are present at approximately 1%, and *Verrucomicrobia* are maintained at low levels, typically less than 1% [24,25]. The gut microbiome characteristics of healthy women exhibit dynamic changes throughout the life cycle and are closely associated with age and sex hormone levels [26]. In infant women, *Proteobacteria* constitute over 50% of the intestinal microbiota, and their proportion gradually decreases, while the related Shannon index demonstrates a significant increase as age advances. In adults, *Firmicutes* and *Bacteroidetes* progressively become predominant, and the Shannon index stabilizes with age [27]. *Faecalibacterium prausnitzii*, *Phocaeicola vulgatus*, and *Bacteroides uniformis* showed significant low numbers in neonatal populations, followed by a gradual enrichment throughout pediatric development until reaching ecological equilibrium in post-adolescent groups. This trajectory starkly contrasts with that of *Bifidobacterium longum*, which displayed peak colonization density during infancy, followed by a progressive decline as development progresses. These changes are associated with the levels of sex hormones, such as estrogen [28,29]. Some researchers have classified the gut microbiota of healthy women into three different enterotypes. Enterotype 1 is defined predominantly by the abundance of butyrate-producing species, such as *Eubacterium rectale* and *Faecalibacterium prausnitzii*. Enterotype 2 is distinguished mainly by the prevalence of lactic acid-producing species, including *Bifidobacterium adolescentis*, *Lactobacillus ruminis*, and *Bifidobacterium bifidum*. Enterotype 3 is marked predominantly by the presence of *Subdoligranulum* spp., *Akkermansia muciniphila*, *Methanobrevibacter smithii*, and *Ruminococcus bromii* [30]. Despite significant differences in gut microbiota characteristics across different races, regions, and individuals [30,31], alterations in the gut microbiota composition can serve as a predictor of certain health issues.

**Vaginal microbiota:** In healthy reproductive-age women, the vaginal microbiota is dominated by *Lactobacillus* spp., including *Lactobacillus crispatus*, *Lactobacillus gasseri*, *Lactobacillus iners*, and *Lactobacillus jensenii* [32]. These bacteria maintain an acidic vaginal environment (pH 3.5–4) by producing lactic acid, inhibiting pathogen colonization. The species diversity of the healthy vaginal microbiota is low. High diversity is often associated with flora disorders such as bacterial vaginosis [33]. To describe its taxonomic composition, the term “community state type” (CST) was introduced [34]. The vaginal microbiota can be briefly divided into five main CSTs according to the subtypes of *Lactobacillus,* including CST I, II, III, V, and CST IV [35]. CST I, II, III, and V are dominated by *Lactobacillus*, while CST IV is characterized by a lower proportion of *Lactobacillus* spp. and a higher proportion of anaerobic bacteria [32,33,36]. CST IV is associated with the development of bacterial vaginosis, which is often accompanied by common gynecological diseases, including sexually transmitted diseases, pelvic inflammatory disease, endometriosis, and gynecological cancers [37,38]. The vaginal microbiota changes dynamically with hormone levels, menstrual cycle, pregnancy, and other physiological stages, but Lactobacillus remains the main component in healthy states [39,40]. Alterations in the vaginal microbiota composition may serve as a potential predictive indicator of gynecological diseases.

### 2.2. Characteristics of the Gut and Vaginal Microbiota in Patients with Ovarian Cancer

Emerging evidence highlights significant microbiome dysregulation in ovarian cancer, with distinct microbial signatures observed in both gastrointestinal and reproductive systems. Clinical investigations consistently demonstrate altered gut microbiota composition in ovarian cancer patients compared to healthy controls.

Key studies revealed decreased α-diversity (Shannon index) and progressive β-diversity reduction during disease progression, particularly in epithelial ovarian cancer (EOC) cohorts [41,42]. α-Diversity measures the microbiota diversity within a single sample and reflects the richness and evenness of a community, while β-diversity measures the diversity between different samples and reflects the differences in diversity between samples or ecosystems [42]. A matched case–control study (*n* = 24/group) identified characteristic taxonomic shifts such as depletion of *Lachnospiraceae*, *Bifidobacteriaceae*, and *Clostridiaceae* families coupled with enrichment in *Coriobacteriaceae* (specifically *Adlercreutzia* and *Collinsella* genera) in cancer patients [43]. These microbial alterations exhibit inter-individual variability potentially influenced by geographic, demographic, and lifestyle factors, yet maintain diagnostic potential through conserved dysbiosis patterns [41,44]. Similar changes were seen in the vaginal microbiome. A multicenter case–control study (*n* = 360) demonstrated that 76.7% of ovarian cancer patients and BRCA1 mutation carriers exhibited a non-*Lactobacillus*-dominated (Type O) vaginal microbiota, contrasting with the Lactobacillus-dominated (Type L) communities prevalent in healthy controls (*p* < 0.05) [19,20]. This ecological shift correlated with decreased *Lactobacillus* abundance, increased microbial diversity, and potential associations with genetic predisposition (e.g., BRCA1 status) and disease pathophysiology [19,41]. Furthermore, longitudinal analyses revealed stage-dependent variations in lower genital tract α-/β-diversity and distinct microbial consortia across ovarian cancer grades [41]. The evidence presented above reveals substantial alterations in both gut and vaginal microbiota among ovarian cancer patients, suggesting a potential link between these microbiota changes and the progression of ovarian cancer.

Notably, specific pathogens including HPV, *Chlamydia trachomatis*, and *Neisseria gonorrhoeae* have demonstrated epidemiological associations with ovarian carcinogenesis [45,46,47]. The collective microbial signatures across anatomical sites show promise as diagnostic biomarkers and may inform future therapeutic strategies. Key clinical findings are systematically summarized in Table 1.

These studies suggest a potential connection between the gut and vaginal microbiomes and ovarian cancer. The collective microbial signatures across anatomical sites demonstrate promising diagnostic potential. Specifically, reproducible alterations including vaginal *Lactobacillus* depletion, enrichment in pathobionts (e.g., *Gardnerella* and *Prevotella*), expansion of gut *Prevotella* and *Escherichia* populations, and reduction in *Bifidobacterium* may serve as valuable auxiliary biomarkers. Given the observed inconsistencies in the α-diversity patterns, robust diagnostic approaches should incorporate multi-parametric features (including microbial taxa, metabolic profiles, and inflammatory mediators) through machine learning integration. The possible mechanisms by which the gut and vaginal microbiomes contribute to the development of ovarian cancer will be described in the following sections.

## 3. Potential Mechanisms of Action of the Gut and Vaginal Microbiomes in Ovarian Cancer

### 3.1. Crosstalk Between Gut and Vaginal Microbiomes

The anatomically adjacent gut and vagina may form a microbiome interaction network. Recent studies have confirmed that the gut and the vaginal microbiota establish a dynamic relationship through various mechanisms, with potential clinical significance for female reproductive health. The main evidence for the bidirectional interaction between gut and vaginal bacteria is described below.

First, the high degree of homology in microbiota composition provides direct evidence for interaction between the two sites. Cross-sectional studies in non-pregnant women showed significant consistency in the distribution of Lactobacillus in the rectum and vagina: the colonization rates of *Lactobacillus crispatus* (16%), *Lactobacillus Jenneri* (10%), and *Lactobacillus gasserii* (10%) in the rectum were highly consistent with their vaginal distribution [48]. This phenomenon was shown to persist during pregnancy, and a study of 132 women at 35 to 37 weeks of gestation found that nearly 50% of bacterial species were detected in the rectum and vagina simultaneously [49]. It is worth noting that Shin et al. [50] confirmed through a longitudinal cohort study that the alpha-diversity index of the two sites showed a convergent trend from the third trimester to 2 months postpartum, suggesting that the perinatal period may enhance cross-site communication between the microbiomes. Especially in patients with CST type IV (non-*Lactobacillus*-dominant) bacterial vaginosis, the rectum has been confirmed to be the reservoir of *Gardnerella* and other pathogenic bacteria, which may lead to a vaginal microecological imbalance through direct migration [51]. These findings suggest a potential association between the components of the gut and vaginal microbiota.

Second, functional intervention experiments revealed cross-organ effects of microbiota regulation. Oral probiotic preparations could significantly change the proportion of vaginal *Lactobacillus* [52] and successfully treat *Gardannella*-induced bacterial vaginitis in animal models [53], which confirms the feasibility of a pharmacological regulation of the “gut–vaginal axis”. Clinical observations have found that the maternal vaginal microbiota affects neonatal intestinal colonization through delivery, a phenomenon called “vaginal seeding”. The abundance of *Bifidobacterium* and other symbiotic bacteria in the gut of infants born by cesarean section is significantly lower than that in newborns born by vaginal delivery. This difference in early flora may increase the risk of long-term obesity [54] and immune-related diseases through metabolic programming [55]. The gut and vaginal microbiota may interact and influence each other, leading to changes in their respective compositions.

Third, molecular mechanistic studies suggest a critical role for estrogen metabolic pathways. The intestinal estrobolome, a functional module of the flora, converts conjugated estrogens into active forms by secreting β-glucuronidase, which enter the systemic circulation through the enterohepatic circulation [55,56,57,58]. High estrogen levels promote *Lactobacillus* proliferation and maintain an acidic environment by up-regulating vaginal glycogen synthesis [59], but the reverse regulation mechanism of the vaginal microbiota has not been elucidated. One of the latest transcriptome studies found that vaginal pathogenic bacteria metabolites may regulate the balance of Th17/Treg in the intestine through the mucosal immune system, but the specific pathway needs to be verified [60]. The detailed mechanisms of the crosstalk between gut and vaginal microbiota remain largely unknown.

Fourth, many phenomena of intestinal and vaginal microecology influencing each other have been observed in clinical practice. The use of antibiotics or probiotics may have a knock-on effect on the gut and vaginal flora. For example, oral probiotics (such as *Lactobacillus*) may indirectly restore the vaginal microbiota balance by improving intestinal barrier function or immune regulation; however, the topical vaginal use of probiotics may affect the gut microbiota by inhibiting pathogen colonization [61,62]. If, after cesarean section infants receive “vaginal seeding” (exposure to maternal vaginal secretions), their gut flora is more similar to that of naturally delivered infants, which demonstrates a bidirectional mother-to-child microbiome transfer [63]. Gut microbiota perturbations (e.g., in inflammatory bowel disease) may disrupt vaginal microbial homeostasis through immune-mediated pathways or metabolic perturbations, elevating the susceptibility to urogenital pathologies such as bacterial vaginosis (BV) and spontaneous preterm birth (sPTB) [64,65]. Conversely, vaginal dysbiosis characterized by *Lactobacillus* depletion may propagate intestinal inflammation via mechanisms involving the translocation of enteric pathogens (e.g., *Escherichia coli)* or the systemic dissemination of pro-inflammatory mediators (e.g., IL-1β, TNF-α) [66]. Notably, the bidirectional crosstalk between these microbial niches demonstrates clinical relevance in metabolic disorders (e.g., obesity-associated dysbiosis) and reproductive malignancies (e.g., cervical carcinogenesis linked to Fusobacterium enrichment) [67]. All these studies suggest that there is an interaction between intestinal and vaginal microecology, but the specific mechanism needs to be further elucidated.

It is concluded that the interaction between intestinal and vaginal microbiota is dynamic and multilayered, involving physical migration, metabolic interaction, immune regulation, and hormone-mediated mechanisms. These interactions not only affect the local microenvironment but also may have profound effects on systemic health through the “entero–vaginal axis”, providing a theoretical basis for the development of intervention strategies targeting the microbiota (e.g., combination probiotics, dietary regulation). With the development of microbial single-cell sequencing and organ-chip technology, re-searchers are expected to systematically reveal the biological significance of the interaction between gut and vaginal flora in the occurrence and development of ovarian cancer and provide a theoretical basis for the further screening of microbial-related markers for the early diagnosis of ovarian cancer.

### 3.2. Estrogen Metabolism Potentiation of the Gut and Vaginal Microbiomes in Ovarian Cancer

#### 3.2.1. Estrogen Metabolism

Estrogen is predominantly synthesized in the ovary, adrenal gland, and adipose tissue and can also be obtained through dietary intake [68]. In the liver, estrogen is converted into conjugated forms via glucuronidation (catalyzed by UDP-glucuronosyltransferase) and sulfation (catalyzed by sulfotransferase), processes that facilitate its biliary excretion or enterohepatic circulation [69]. Circulating estrogen exists primarily in three forms, i.e., estrone, estradiol, and estriol, which may be either free or protein-bound [70]. Estrogens exert their biological effects by binding to classical nuclear receptors (estrogen receptors α and β, ER-α, and ER-β) or non-classical membrane receptors (G protein-coupled estrogen receptor, GPER) [71,72]. Additionally, the intestinal microbiota plays a role in the enterohepatic circulation of estrogen by metabolizing estrogen and its metabolites, thereby regulating physiological activities upon reabsorption [73]. The microbiota is an essential part that influences human estrogen metabolism.

#### 3.2.2. Epidemiological Evidence and Mechanisms of the Association Between Estrogen and Ovarian Cancer

The current epidemiological evidence supports estrogen exposure (especially long-term MHT use) as an independent risk factor for ovarian cancer. A case–control study involving 800 black women cases and 1783 controls, as well as 2710 white women cases and 8556 controls, was conducted to evaluate the association between menopausal hormone therapy (MHT) and the risk of ovarian cancer. Long-term MHT (≥10 years) was found to be significantly associated with an increased risk of ovarian cancer among white women (OR = 1.38, 95% CI: 1.22–1.57). This finding was consistent in black women [74]. The long-term use of unopposed estrogen (e.g., continuous use for over 5 years) is particularly linked to elevated risks of high-grade serous and endometrioid tumors [75]. However, in contrast to estrogen alone, a serial combination therapy with estrogen and progestin (e.g., medroxyprogesterone acetate) did not significantly increase the risk of ovarian cancer overall (OR = 0.85, 95% CI = 0.72–1.0) and may even reduce the risk of mucoid ovarian cancer (OR = 0.40, 95% CI = 0.18–0.91) [76]. A two-sample Mendelian randomization (MR) study also suggested that higher endogenous estrogen levels (estradiol levels) (OR = 3.18, 95% CI = 1.47–6.87) were an independent risk factor for ovarian cancer [77]. Epidemiological evidence shows that excessive estrogen is a risk factor for ovarian cancer.

**Figure 2:**
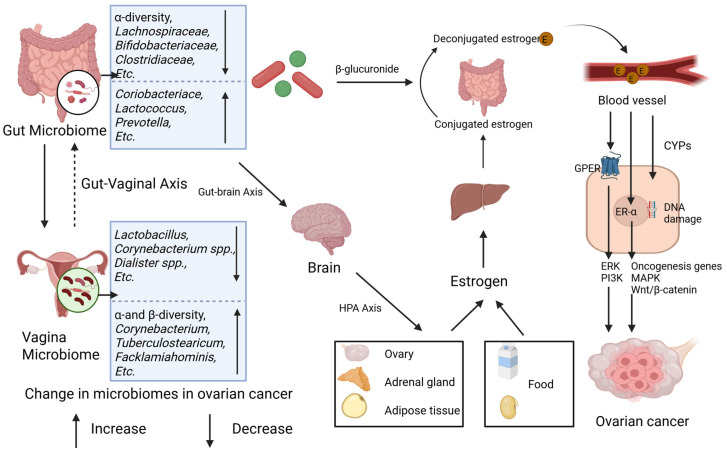
Gut and vaginal microbiomes and estrogen in ovarian cancer. Gut and vaginal microbiomes interact with each other via gut–vaginal axis. Gut and vaginal microbiomes change in patients with ovarian cancer. Gut microbiome changes circulatory estrogen levels by estrobolome and gut–brain axis. Estrobolome increases the levels of circulating estrogen by β-glucuronides. Gut–brain axis leads to stress and then activates hypothalamic–pituitary–adrenal (HPA) axis, regulating generation of estrogen. Excessive estrogen combines with ER-α and G-protein-coupled estrogen receptor (GPER) and then activates signaling pathways including ERK, PI3K, etc., and oncogenesis genes. Meanwhile, metabolites of estrogen, via CYPs, elevate level of free radicals causing DNA damage and promoting tumorigenesis.

Excessive estrogen may stimulate the proliferation of ovarian epithelial cells and expedite the progression of ovarian cancer via receptor-dependent pathways (e.g., ER-α and GPER pathways) as well as receptor-independent mechanisms (e.g., cytochrome P450 enzyme, CYP450 pathways) [78]. Upon binding to estrogen, ER-α can trigger the activation of downstream oncogenes (such as c-fos, c-myc, and HER2/neu) and modulate mitogen-activated protein kinase (MAPK) and Wnt/β-catenin signaling pathways, which are intricately associated with ovarian carcinogenesis [79,80,81,82]. GPER facilitates cancer cell proliferation by activating second messenger systems, including extracellular signal-regulated kinase (ERK) and phosphatidylinositol-3-kinase (PI3K) [83]. Moreover, estrogen metabolites (e.g., quinone intermediates) can induce gene mutations by elevating the free radical levels through the action of CYP450 enzymes [21] (Figure 2).

#### 3.2.3. Microbiome–Estrogen-Mediated Ovarian Cancer

The gut microbiota influences estrogen homeostasis through two principal pathways—the estrobolome and the gut–brain axis—with potential implications for ovarian carcinogenesis (Figure 2). The estrobolome, a microbial network dominated by Firmicutes and Bacteroidetes, governs estrogen metabolism via β-glucuronidase activity [56,84]. This enzyme hydrolyzes conjugated estrogens, enabling the enterohepatic recirculation of free estrogen and elevating the systemic estrogen levels, a mechanism implicated in ovarian cancer pathogenesis [78,79]. Parallelly, gut-derived metabolites (e.g., γ-aminobutyric acid [GABA], norepinephrine) engage the gut–brain axis by stimulating vagal afferents and central neurons [85]. These interactions activate the hypothalamic–pituitary–adrenal (HPA) axis, linking neuroendocrine stress responses (e.g., anxiety, depression) to gonadal hormone dysregulation and subsequent estrogen fluctuations [86,87,88]. In contrast, the vaginal microbiome contributions to estrogen modulation remain poorly characterized.

The gut–vaginal axis facilitates the bidirectional crosstalk between intestinal and vaginal microbial communities. Intestinal dysbiosis disrupts estrogen homeostasis via two mechanisms: (1) the estrobolome-mediated hydrolysis of conjugated estrogens; (2) the gut–brain axis-driven activation of the HPA axis, which alters gonadal hormone synthesis. Elevated estrogen levels promote ovarian carcinogenesis through receptor-dependent proliferation of malignant cells and receptor-independent DNA damage by genotoxic metabolites [21]. These synergistic mechanisms collectively drive oncogenesis and disease progression.

These findings suggest a potential association between the gut and vaginal microbiota and estrogen metabolism, indicating that microbiota dysbiosis may contribute to the development of ovarian cancer.

### 3.3. Chronic Inflammatory Priming by the Gut and Vaginal Microbiomes in Ovarian Cancer

**Figure 3:**
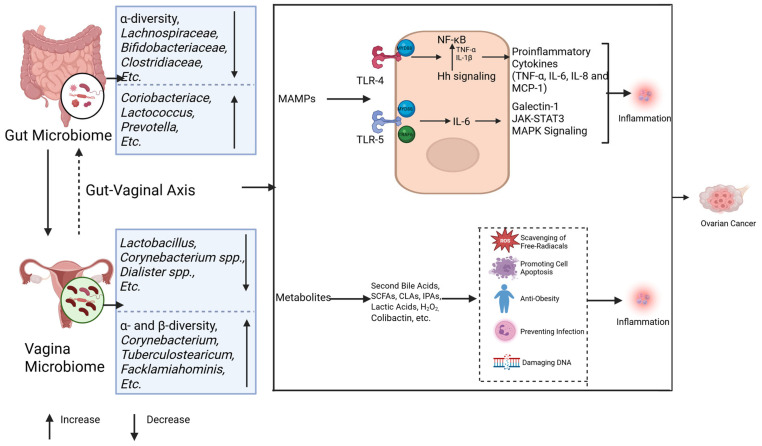
Microbiomes and inflammation in ovarian cancer. The gut and vaginal microbiomes interact with each other via the gut–vaginal axis. The gut and vaginal microbiomes change in patients with ovarian cancer. The gut and vaginal microbiomes are associated with inflammation mainly through MAMPs and metabolites. MAMPs interact with TLR-4 and TLR-5, activating their downstream signaling and leading to tumor development by promoting inflammation. The metabolites of microbes can inhibit the inflammatory reaction, while some of them (colibactin) cause DNA damage and promote tumor genesis.

The dual role of inflammation in tumorigenesis has been well established through extensive research. Acute inflammation exerts anti-tumor effects via dendritic cell activation and enhanced antigen presentation, whereas chronic inflammation fosters a tumor-promoting microenvironment [89]. Prolonged acute inflammation may transition into chronic inflammation, sustaining inflammatory signaling pathways while establishing hypoxic and acidic conditions. This pathological microenvironment facilitates immunosuppressive cell infiltration and the activation of oncogenic pathways, ultimately driving DNA damage and neoplastic transformation [90]. Notably, established ovarian cancer risk factors including psychological stressor and obesity share common pro-carcinogenic mechanisms through inflammatory mediators such as interleukins and interferons [9,91,92]. Furthermore, inflammatory responses serve as the principal pathway through which microbial organisms and their metabolic byproducts contribute to ovarian carcinogenesis [60,93,94] (Figure 3). The main pathways by which gut and vaginal microbes influence the development and progression of ovarian cancer through inflammatory responses will be described below.

#### 3.3.1. The Gut Microbiota Influences Ovarian Cancer Development Through Inflammation

Host pattern recognition receptors (PRRs) are essential in microbiota-associated inflammation. The intestinal microbiota initiates inflammatory responses through microbe-associated molecular patterns (MAMPs) that engage host PRRs. In ovarian cancer, Toll-like receptors 4 (TLR4) and 5 (TLR5) represent the most prominently implicated PRRs (Figure 3).

Lipopolysaccharide (LPS) derived from the gut microbiota activates the TLR4/NF-κB signaling cascade via the myeloid differentiation factor 88 (MYD88) adaptor. This pathway upregulates proinflammatory cytokines—including TNF-α, IL-6, IL-8, and MCP-1—while concurrently stimulating anti-apoptotic mechanisms. Together, these effects drive ovarian carcinogenesis by enhancing angiogenic signaling and cell survival programs [95,96,97]. Notably, TLR4 exhibits functional crosstalk with the hedgehog (Hh) signaling pathway. Preclinical studies demonstrate that the ovarian cancer-associated gut microbiota accelerates tumor proliferation through sonic hedgehog (Shh)/Gli-1 axis activation, whereas Hh inhibitors (e.g., GANT61) suppress tumorigenesis [42,98]. Mechanistically, NF-κB amplifies cancer cell invasiveness by elevating TNF-α and IL-1β production, which subsequently enhances Shh promoter activity and Gli-1 expression [99]. Parallel TLR5 signaling through the MyD88/TRAF6 complex elevates IL-6 production and induces galectin-1 secretion via myeloid-derived suppressor cells (MDSCs) and γδ T cells, collectively fostering tumor progression [18]. Critically, IL-6 activates the JAK-STAT3 and MAPK pathways—key drivers of metastasis and poor clinical outcomes in patients with high-grade ovarian malignancies [100]. MAMPs can trigger PRR-associated chronic inflammation, thereby promoting the development of ovarian cancer.

**Gut microbial metabolites** (Figure 3): Gut microbial metabolites, such as secondary bile acids, short-chain fatty acids (SCFAs), indolepropionic acids (IPA), and genotoxic metabolites, also have an important impact on ovarian cancer occurrence through different pathways [22]. These metabolites act locally or distally through the circulatory system to inhibit inflammation, scour free radicals, and promote apoptosis [93,94]. For example, deoxycholic acid (DCA) in secondary bile acids induces apoptosis of ovarian cancer cells (such as A2780 and A2780-CP) through a PKC-independent pathway, showing anti-tumor effects [101,102]. SCFAs, such as acetate, propionate, and butyrate, are produced by the fermentation of undigested carbohydrates, inhibit the NF-κB signaling pathway, and promote macrophage differentiation, thereby inhibiting tumorigenesis [103,104]. IPA plays an anti-tumor role by scavenging free radicals, and the IPA levels are reduced in ovarian cancer patients [105,106]. Dysbiosis of the gut microbiota can promote tumorigenesis through inflammation and DNA damage by producing genotoxic metabolites, such as colibactin [107,108]. These results strongly suggest that dysbiosis of the gut microbiota promotes ovarian cancer by inducing chronic inflammation. However, the contributions of specifically altered microbiota in ovarian cancer still warrant further investigation.

#### 3.3.2. Vaginal Microbes Influence Ovarian Cancer Development Through Inflammation

*Lactobacillus* is the main commensal bacteria of the vaginal microbiome and inhibits pathogen infection by producing lactic acid and H_2_O_2_ [48]. Studies have shown that Lactobacillus vaginalis can induce apoptosis of CAOV-4 cells and inhibit the development of ovarian cancer by down-regulating miR-21, miR-200b, and TLR4 [109]. When the vaginal flora is dysregulated, pathogens such as Chlamydia trachomatis and human papillomavirus (HPV) can inhibit cell apoptosis by blocking caspase 3 and cytochrome C release and activate TLRs in epithelial cells to promote the occurrence of ovarian cancer [110,111]. In addition, pathogen infection also promotes ovarian cancer development by regulating DNA damage repair, p53 inactivation, and MAPK signaling pathways [110] (Figure 3).

Additionally, clinical research has found that ovarian cancer patients commonly exhibit co-enrichment of LPS-producing Gram-negative bacteria in both gut and vaginal microbiota, creating a systemic inflammatory state that promotes tumor metastasis via the IL-6/STAT3 signaling pathway [112], suggesting that the vaginal microbiota promotes tumorigenesis by chronic inflammation activation.

In conclusion, the gut and vaginal microbiota significantly affect the development of ovarian cancer through inflammation-related mechanisms, such as the interaction between MAMPs and PRRs, the regulation of metabolites, and pathogen infection. These mechanisms involve the activation of proinflammatory signaling pathways, alterations in the immune microenvironment, and accumulation of DNA damage. The microbiota–inflammation axis can be used as a biomarker for the early diagnosis of ovarian cancer (such as the LPS/IL-6 ratio in vaginal fluid) and as a new therapeutic target.

### 3.4. Epigenetic Modifications by the Gut and Vaginal Microbiomes and Epigenetics in Ovarian Cancer

#### 3.4.1. Epigenetic Modifications and Ovarian Cancer

The genetic factor is an essential risk factor for ovarian cancer. Epigenetic modifications, primarily including DNA methylation, histone modifications, and non-coding RNAs, serve as pivotal mediators bridging genetic susceptibility and environmental exposures in ovarian carcinogenesis. These modifications dynamically regulate gene expression without altering DNA sequences, enabling the integration of external stimuli (such as microbial metabolites and inflammatory signals) with intrinsic genetic programs. Through reversible epigenetic mechanisms, they coordinate genetic and environmental factors to drive ovarian cancer progression. Specifically, the dysregulation of DNA methylation, histone modifications, and non-coding RNA-mediated gene silencing leads to aberrant gene expression, disrupting the balance of cell proliferation and apoptosis while promoting the remodeling of the tumor microenvironment. Collectively, these epigenetic alterations act as critical drivers of oncogenesis by integrating environmental cues with genetic predispositions to foster malignant transformation.

**DNA Methylation Alterations in Ovarian Cancer:** DNA methylation is the most extensively studied epigenetic mechanism in ovarian cancer. A genome-wide methylation analysis has revealed a large number of abnormally hypermethylated or hypomethylated regions in ovarian cancer; for example, hypomethylation of the POTEE gene promoter is strongly associated with the development of ovarian cancer [113]. These methylation alterations can drive tumorigenesis through the silencing of tumor suppressor genes (e.g., BRCA1/2) or the activation of oncogenes [114,115]. In addition, the DNA methylation status can be used as an early diagnostic marker for ovarian cancer; for example, the methylation patterns of specific genes in blood have been used in liquid biopsy development [115].

**Histone Modifications and Chromatin Remodeling in Ovarian Cancer:** Histone methylation (such as EZH2-mediated H3K27me3) and deacetylation (HDAC-mediated) affect gene expression by regulating chromatin structure. Studies have found that EZH2 is highly expressed in ovarian cancer and enhances tumor invasiveness by inhibiting the MAF gene [116]. Aberrant expression of CHD4, a chromatin helicase DNA-binding protein, is associated with metastasis and may be a therapeutic target [117,118]. Targeting histone modifications and chromatin remodeling may be a potential therapy for ovarian cancer.

**Non-coding RNA Regulation in Ovarian Cancer:** MicroRNAs (miR-203a-3p, etc.) and long non-coding RNAs (such as OIP5-AS1) regulate ZEB1 and other epithelial–mesenchymal transition-related genes through epigenetic mechanisms to promote ovarian cancer metastasis [119]. m6A RNA methylation modification has been found to dynamically regulate RNA metabolism, and its abnormal levels are closely associated with ovarian cancer drug resistance [120]. The detection of non-coding RNAs holds the potential to assess ovarian cancer development and therapeutic efficacy.

Given their widespread observation in ovarian cancer, specific epigenetic modifications hold potential as diagnostic markers or predictors of the therapeutic response.

#### 3.4.2. Epigenetic Regulation by the Gut Microbiota in Ovarian Cancer

**Metabolites of the gut microbiota mediate epigenetic modifications:** The gut microbiota can produce metabolites such as SCFAs, butyric acid, and propionic acid by fermenting dietary fiber. These metabolites can directly inhibit histone deacetylases (HDACs), leading to an increase in histone acetylation levels. This activates the expression of tumor suppressor genes (such as P21 and P53) and inhibits the proliferation of ovarian cancer cells [121,122]. In addition, LPS and secondary bile acids produced by certain pathogenic bacteria (such as *Escherichia coli*) may induce the abnormal methylation of oncogenes (such as MYC) through DNA methyltransferases (DNMTs), promoting the progression of ovarian cancer [44,123]. Moreover, folate and some B vitamins produced by the gut microbiota can participate in the methylation of DNA and histones by providing methyl groups [123]. These findings indicate that metabolites produced by the gut microbiota may mediate epigenetic modifications associated with ovarian cancer.

**Gut microbiota-mediated epigenetic modifications:** The gut microbiota can activate the Toll-like receptor (TLR) signaling pathway of the host immune cells (such as dendritic cells and T cells) through metabolites or cell components (such as peptidoglycan) and then regulate DNA methylation and histone modification, affecting the expression of immune checkpoint molecules (such as PD-1 and CTLA-4). For example, *Bifidobacterium* can enhance the histone H3K27 acetylation level in CD8+ T cells and enhance their anti-tumor activity, while pathogenic bacteria may inhibit immune responses through DNMTs to form an immunosuppressive microenvironment [123,124,125]. The gut microbiota can regulate the expression of host RNA m6A (N6-methyladenosine) modification enzymes (such as METTL3 and FTO), affecting the translation efficiency and stability of ovarian cancer-related genes. For example, a gut microbiota imbalance may reduce the activity of m6A reader proteins (such as YTHDF2), leading to enhanced mRNA stability of oncogenes (such as VEGF and MMP9), thereby promoting tumor angiogenesis and metastasis [126]. The gut microbiota can modulate DNA methylation and the expression of non-coding RNAs (e.g., miRNAs) in host genes by secreting small RNAs (sRNAs) or extracellular vesicles (EVs), which are directly delivered to the host ovarian cells. For instance, sRNAs derived from *Fusobacterium nucleatum* may suppress the transcription of tumor suppressor genes such as BRCA1 while promoting the epigenetic activation of pro-inflammatory genes like IL-6, thereby accelerating the progression of ovarian cancer [124,127]. In addition, aging-induced alterations in the gut microbiota (e.g., a decreased *Firmicutes*/*Bacteroidetes* ratio) may induce telomere shortening and the accumulation of DNA methylation senescence markers (e.g., p16INK4a) in ovarian cells through oxidative stress pathways, while inhibiting the differentiation capacity of ovarian stem cells and accelerating the risk of ovarian decline and cancer [128,129]. As previously discussed, alterations in the gut microbiota are associated with epigenetic modifications.

#### 3.4.3. Epigenetic Regulation by the Vaginal Microbiota in Ovarian Cancer

**Metabolites of the vaginal microbiota mediate epigenetic modifications:** SCFAs such as butyric acid produced by some vaginal symbiotic bacteria (such as lactic acid bacteria) can increase the level of histone acetylation by inhibiting histone deacetylases (HDACs), thereby activating the expression of tumor suppressor genes [112,122]. LPS released by Gram-negative bacteria (such as Gardnerella vaginalis) induces the secretion of pro-inflammatory factors (such as IL-6 and TNF-α) by activating the Toll-like receptor (TLRs) signaling pathway, which results in the abnormal activity of DNA methyltransferases (DNMTs) that in turn silence tumor suppressor genes (such as BRCA1) or activate oncogene [112]. Studies have shown that abnormal methylation of BRCA1/2 genes in ovarian cancer tissue is closely associated with microbial-associated inflammation [114], further demonstrating the ability of vaginal microbiota metabolism to promote ovarian cancer development by epigenetic modifications.

**Vaginal microbiota-mediated epigenetic modifications:** *Chlamydia trachomatis* infection can induce abnormal DNA methylation in host cells, leading to the inactivation of tumor suppressor genes such as PTEN [130,131]. Mycobacterium interferes with histone modification by secreting effector proteins, activates pro-cancer pathways (such as Wnt/β-catenin), and promotes the malignant transformation of ovarian epithelial cells [132].

**Epigenetic reprogramming driven by the inflammatory microenvironment:** Vaginal microbial imbalances (e.g., non-lactic acid bacteria-dominated communities) can trigger chronic inflammation and indirectly affect epigenetic regulation. After activating the NF-kappa B pathway, inflammatory factors can recruit DNA methylases or histone-modifying enzymes to specific gene promoter regions. For example, persistent stimulation of IL-1β in ovarian cancer cells can lead to hypermethylation and loss of expression of the tumor suppressor gene PTEN [124]. The accumulation of reactive oxygen species (ROS) caused by microbial dysregulation can interfere with the histone acetylation/methylation balance and promote cancer-promoting chromatin remodeling. Animal models show that vaginal flora disturbance can exacerbate ovarian tissue oxidative damage and epigenetic abnormalities [132]. This indicates that the vaginal microbiota can also indirectly regulate ovarian cancer-related epigenetic modifications through inflammation.

**Non-coding RNA regulation in microbial–host interactions:** Vaginal microbes may influence ovarian carcinogenesis by regulating host non-coding RNAs, such as miRNAs. For example, a decrease in lactic acid bacteria is associated with the upregulation of miR-21 (cancer-promoting miRNA), which promotes the proliferation of ovarian cancer cells by inhibiting target genes such as PTEN [112,114]. Studies suggest that the vaginal flora can pass microbial RNA or regulate the host miRNAs through exosomes, affecting the epigenetic state of ovarian epithelial cells [22,127]. In ovarian cancer, the abnormal expression of m6A modification enzymes (such as METTL3) is related to microbiota disorders. Microbial metabolites may affect the stability and translation efficiency of carcinogenesis-related mRNAs by regulating the activity of m6A modification enzymes [133]. Non-coding RNAs are key mediators through which the vaginal microbiota promotes ovarian carcinogenesis.

**Epigenetic remodeling of immune cells:** The vaginal microbiome indirectly affects ovarian cancer microenvironment by modulating immune cell function. A specific vaginal flora (e.g., *Preveria*) can induce the demethylation of the FOXP3 gene in Treg cells, promoting the formation of an immunosuppressive microenvironment and accelerating the immune escape of ovarian cancer [112,134]. Microbial-related metabolites (such as tryptophan derivatives) promote M2-type macrophage polarization by inhibiting histone deacetylation, and these macrophages in turn secrete factors such as IL-10, supporting tumor growth [112,132]. These findings highlight the critical role of the vaginal microbiota in promoting ovarian cancer progression by immune cell-associated epigenetic remodeling.

In fact, the vaginal and gut microbiota may synergistically influence the ovarian epigenetic status through the “vaginal–gut axis”. Secondary bile acids produced by the intestinal flora can reach the ovaries through the blood circulation, regulate DNA methylation by activating FXR receptors, and promote the self-renewal of ovarian cancer stem cells [122,132]. An imbalance in the vaginal flora can lead to increased beta-glucuronidase activity and promote estrogen resorption, and estrogen can induce DNA methyltransferase expression by binding its receptors, driving the progression of hormone-dependent ovarian cancer [130]. Bacteria causing intestinal flora imbalances (e.g., *Bacteroidetes*) may migrate to the genital tract through the lymphatic system or blood circulation, forming a pro-inflammatory microenvironment with the vaginal flora, activating epigenetic regulatory networks in macrophages and T cells (e.g., miRNA-mediated immune checkpoint gene silencing), and accelerating ovarian cancer progression [135]. Current research has indicated the correlation between the gut and vaginal microbiota and epigenetic modifications in ovarian cancer. However, the specific alterations in microbial species and their relationship with epigenetic modifications at different stages of ovarian cancer remain to be further explored.

## 4. Discussion

The prediction and early diagnosis of ovarian cancer are far from satisfactory. Unclear pathogenesis and delayed diagnosis in ovarian cancer urgently need to be solved. Recent advances in microbiome research suggest the potential of the microbiota for the diagnosis and management of human disease [131] and offer us an entirely new way to better understand the mechanisms of ovarian cancer development and the opportunity to optimize the prevention and early diagnosis of ovarian cancer.

As outlined above, extensive animal and human studies have demonstrated that ovarian cancer patients exhibit unique intestinal and vaginal microbiota and associated metabolites. The mechanisms by which these microorganisms and their metabolites contribute to the development of ovarian cancer have been elucidated to some extent [136]. Several Mendelian randomization studies conducted in European populations suggest that certain microbial genera, such as *Lachnospiraceae*, *Oscillospira*, *Bifidobacterium*, and *Alistipes*, may reduce the risk of ovarian cancer. Conversely, *Bacteroides*, *Ruminococcus*, *Lactobacillus*, and *Prevotella* are associated with an increased risk of ovarian cancer [137,138,139]. These findings reinforce the causal relationship between the gut microbiota and ovarian cancer and provide a foundation for identifying and utilizing early biomarkers for ovarian cancer screening. Despite limited research on leveraging these differential microorganisms, metabolites, and metabolic pathways for diagnosing ovarian cancer, the results obtained so far offer novel approaches for screening and applying such microbiota-related features as biomarkers. Cheng Chen et al. [44], based on a small sample population (34 patients diagnosed with epithelial ovarian cancer (EOC), 15 patients with benign ovarian tumors (BOTs), and 30 healthy volunteers (NOR)), identified 11 intestinal microbes strongly associated with epithelial ovarian cancer. Among them, *Shigella*, *Escherichia*, *Haemophilus*, and *Bifidobacterium* were used in their diagnostic model. Using Least Absolute Shrinkage and Selection Operator (LASSO) analysis and support vector machines (SVMs), they developed a diagnostic model for ovarian cancer with an area under the curve (AUC) value of 0.709 (95% CI: 0.595–0.824) based on the receiver operating characteristic (ROC) curve. Zhang L. et al. [138] constructed a diagnostic model for ovarian cancer using vaginal microbial diversity and microbiota characteristics, including the significant abundance of Lactobacillus and the enrichment in Gardnerella and Prevotella, achieving an AUC of 0.83, sensitivity of 76%, and specificity of 77%. This finding indicates that relying solely on the gut or vaginal microbiota can enhance the diagnostic accuracy of ovarian cancer. Currently, most of these studies are observational and involve small sample sizes without distinguishing ovarian cancer subtypes; therefore, prospective cohort studies are necessary to validate marker stability. Given that the microbiota characteristics are closely linked to factors such as region, race, age, diet, and environment, integrating metagenomic, metabolomic, and epigenetic data is essential for exploring and evaluating more specific biomarkers for targeted populations. In the future, combining gut and vaginal microbial profiles, their metabolites, and oncogenic pathway-related markers holds promise for further improving the accuracy of early ovarian cancer diagnosis.

It is well known that ovarian cancer is a complex chronic disease whose pathogenesis has not been fully elucidated, and its occurrence is associated with many factors including environmental factors [9], genetic factors [8,140], demographic and lifestyle factors [10], reproductive factors [11], and microbiome factors [20,44] (Figure 1). Although recent research revealed the separate effects of these risk factors, their cooperation in individuals has been almost ignored, which may be a blockage in exploring the development of ovarian cancer. These risk factors exert different effects in specific patients with ovarian cancer. We need to make full use of modern molecular biology technology, especially high-throughput multi-omics approaches, operating at the molecular level to comprehensively screen and identify ovarian cancer biomarkers considering the aspects of microbiology, metabolomics, genomics, transcriptomics, key signaling pathways, and tissue target cells. Concurrently, elucidating the interactions between these factors is essential for achieving both mechanistic insights and the discovery of high-specificity biomarkers. Epidemiological frameworks for chronic disease causality (e.g., web of causation, ecological models) should be used [139]. In addition to microorganisms and their metabolites, related-pathway genes, epigenetic modification products, a series of clinical symptoms, serological and other indicators are also factors related to ovarian cancer. The comprehensive use of these indicators is very important for the construction of a high-accuracy early-diagnosis model of ovarian cancer with big data analysis methods such as machine learning and multimodal processing [141,142]. We therefore propose the following future goals: defining the interactions between the intestinal/cervicovaginal microbiota and ovarian cancer through multi-etiological epidemiological models to identify novel biomarkers; and leveraging multimodal data integration and machine learning [143,144] to unify traditional risk factors with molecular markers and develop high-sensitivity and -specificity early diagnostic models through these synergistic approaches.

## 5. Conclusions

In conclusion, ovarian cancer patients exhibit distinct gut and vaginal microbiota profiles, with interactions occurring between these microbial communities. The gut and vaginal microbiomes may influence the progression of ovarian cancer through mechanisms such as estrogen regulation, modulation of inflammatory and immune responses, and epigenetic modifications. Relevant markers have demonstrated potential application value in the early diagnosis of ovarian cancer; however, there is a lack of prospective validation using large sample sizes across diverse populations. Given that diagnostic delays significantly contribute to ovarian cancer mortality, it is feasible to construct a more accurate early-diagnosis model by integrating multiple etiological factors from epidemiology research, utilizing multimodal approaches and machine learning techniques. This model could incorporate intestinal and vaginal microbial markers, genetic factors, clinical test results, and other indicators. Such an approach represents a critical direction for future research.

## Figures and Tables

**Table 1:** Characteristics of the gut and vaginal microbiomes in patients with ovarian cancer.

Study	Study Design	Participants	Associated Outcomes
Nené 2019 [19]	Case–control study	EOC patients (*n* = 176) Healthy controls (*n* = 115) Benign gynecological conditions (*n* = 69)	Vaginal microbiota: Higer rate of CST O in EOC group and BCRA1 mutation group Carrying rate of CST O is associated with age and family history
Jacobson 2021 [20]	Cohort Study	Primary platinum-resistant EOC patients (*n* = 17) Platinum-super-sensitive EOC patients (*n* = 23) Benign-gynecologic-condition patients (*n* = 5)	Gut microbiota: Patients with ovarian cancer had significantly higher relative abundance of *Prevotella* in the gut microbiome compared to individuals with benign conditions Vaginal microbiota: Approximately 24% (11 of 45) of patients in this study had *Lactobacillus*-dominated communities, which was significantly lower compared with the results of studies of similarly aged women without ovarian cancer
Asangba 2023 [41]	Cohort study	OC (*n* = 30) Benign gynecologic condition (*n* = 34)	Vaginal microbiota: Increase in α- and β-diversity in patients with ovarian cancer compared with individuals with benign gynecologic conditions Increase in *Corynebacterium*, *tuberculostearicum*, *Facklamiahominis,* and *Ruminococcus*; Decrease in *Corynebacterium* spp. and *Dialister* spp.
Hu 2023 [42]	Case–control study	EOC patients (*n* = 20), EBOT patients (*n* = 20) Healthy (*n* = 20)	Gut microbiota: Decrease in Shannon index and α-diversity Decrease in relative abundance of *Actinobacteria* phyla, *Bifidobacterium*, and *Ruminococcaceae* (*Ruminococcus*) Increase in relative abundance of *Proteobacteria phyla*, *Bacteroides*, and *Prevotella*
D’Amico 2021 [43]	Cohort study	EOC patients (*n* = 24) Healthy controls (*n* = 24)	Gut microbiota: Decrease in Lachnospiraceae, Bifidobacteriaceae, Clostridiaceae, Rikenellaceae, and Porphyromonadaceae Increase in Coriobacteriace (Adlercreutzia and Collinsella) Lactococcus, and Lachnobacterium in ovarian cancer patients compared to healthy controls Difference in α-diversity was not observed
Chen C. 2025 [44]	Case–control study	EOC patients (*n* = 34) Benign ovarian tumors (TB) (*n* = 15) Healthy volunteers (*n* = 15)	Gut microbiota: Decrease in α-diversity indices (Chao1 and Shannon index) Decrease in abundance of *Bilophila, Bifidobacterium,* and other probiotics in patients with EOC Increase in *Escherichia* and *Shigella* in patients with EOC

Abbreviations: OC: ovarian cancer; EOC: epithelium ovarian cancer; EBOT: epithelial benign ovarian tumor; TB: benign ovarian tumors.

## Data Availability

No new data were generated or analyzed in this review.
